# Prognostic Analysis of Human Pluripotent Stem Cells Based on Their Morphological Portrait and Expression of Pluripotent Markers

**DOI:** 10.3390/ijms232112902

**Published:** 2022-10-26

**Authors:** Olga A. Krasnova, Vitaly V. Gursky, Alina S. Chabina, Karina A. Kulakova, Larisa L. Alekseenko, Alexandra V. Panova, Sergey L. Kiselev, Irina E. Neganova

**Affiliations:** 1 Institute of Cytology, 194064 Saint Petersburg, Russia; 2 Ioffe Institute, 194021 Saint Petersburg, Russia; 3 Endocrinology Research Centre, 115478 Moscow, Russia; 4 Vavilov Institute of General Genetics, Russian Academy of Sciences, 117971 Moscow, Russia

**Keywords:** human embryonic stem cells, human induced pluripotent stem cells, morphological parameters, pluripotency markers, classification

## Abstract

The ability of human pluripotent stem cells for unlimited proliferation and self-renewal promotes their application in the fields of regenerative medicine. The morphological assessment of growing colonies and cells, as a non-invasive method, allows the best clones for further clinical applications to be safely selected. For this purpose, we analyzed seven morphological parameters of both colonies and cells extracted from the phase-contrast images of human embryonic stem cell line H9, control human induced pluripotent stem cell (hiPSC) line AD3, and hiPSC line HPCASRi002-A (CaSR) in various passages during their growth for 120 h. The morphological phenotype of each colony was classified using a visual analysis and associated with its potential for pluripotency and clonality maintenance, thus defining the colony phenotype as the control parameter. Using the analysis of variance for the morphological parameters of each line, we showed that selected parameters carried information about different cell lines and different phenotypes within each line. We demonstrated that a model of classification of colonies and cells by phenotype, built on the selected parameters as predictors, recognized the phenotype with an accuracy of 70–75%. In addition, we performed a qRT-PCR analysis of eleven pluripotency markers genes. By analyzing the variance of their expression in samples from different lines and with different phenotypes, we identified group-specific sets of genes that could be used as the most informative ones for the separation of the best clones. Our results indicated the fundamental possibility of constructing a morphological portrait of a colony informative for the automatic identification of the phenotype and for linking this portrait to the expression of pluripotency markers.

## 1. Introduction

Pluripotent stem cells are capable of unlimited proliferation and self-renewal. The application of human pluripotent stem cells (hPSCs), including human embryonic stem cells (hESCs) and human induced pluripotent stem cells (hiPSCs), is intended to make a revolutionary breakthrough in the field of regenerative medicine, the creation of new drugs, the in vitro modeling of human diseases, and cell bank development [1,2,3,4]. In our study, we focused on the morphological characterization of genetically different three hPSC lines, one of which (H9, WiCell) is the most widely used in worldwide research, while the other two comprise control hiPSC line AD3 [5] and patient-specific hiPSC line HPCASRi002-A (CaSR), further referred to as hiPSC line CaSR [6].

Under in vitro conditions, hPSCs grow in the form of monolayer multicellular colonies with characteristic morphological features. The use of hPSCs in clinic requires a high level of quality control; a homogeneous, clonal composition of the cell population is one of the necessary conditions for their safe application. Various molecular methods are used to assess the state of undifferentiated, healthy colonies. However, the use of invasive assessment methods does not allow these cells to be further used in clinical practice. The noninvasive assessment of cell culture quality is currently not quantitative and can often be biased. It may depend on the experience of the investigator or subtle morphological differences between cell lines and clones. The various existing commercial high-content/high-throughput image acquisition systems not always can be applicable for the morphological analysis of hPSC colonies, since multicellular colonies are formed by close-packed small cells (10–16 μm in diameter), many parameters of which may be overlooked during automatic image analyses [7,8,9,10]. Thus, a computer analysis of the colony image should be specific for hPSCs, since otherwise it may fail to notice the differences in the morphological features of the cells and colonies, which often reflect the beginning of the differentiation process.

Clonality is another important characteristic of hPSC colonies. The clonal origin of hiPSC colonies guarantees their further safe use in the clinic, since the heterogeneous composition of the colony is directly related to adaptation in culture leading to the occurrence of chromosomal aberrations [7]. The loss of clonality leads to a large variability in clones not only at the level of pluripotency marker expression but also in terms of their ability to efficiently target differentiation, leading to the emergence of mutations [11,12,13]. It was shown on three hESC lines that colonies of clonal composition formed embryoid bodies (EBs) much less successfully than colonies of nonclonal origin [14]. In vivo (formation of teratomas) and in vitro (formation of EBs) methods for analyzing the quality of hPSCs are not necessarily reliable criterion and measurement of a healthy (clonal) hPSC culture, since they can assess the development potential of individual self-renewing subclones and cannot be sensitive to its nonclonal composition [14]. The molecular basis of the heterogeneity of hPSC colonies has been given little attention and is mainly represented by data about the heterogeneous expression of numerous transcription factors [15].

Many studies focused on studying the genetic, epigenetic, molecular, and signaling pathways that control the induction and maintenance of pluripotency, as well as the mechanisms of hPSC self-renewal. At the same time, how a stem cell colony is formed, how its growth and cell behavior are regulated, and how this is related to the morphological and functional parameters remain poorly understood. It is important to emphasize that the parametric analysis of the morphological characteristics of various hiPSC clones is in good agreement with the gene expression data; thus, it is a reliable indicator of their quality [16,17]. These works confirmed that using an unlabeled method of image analysis, the quality of the clones could be assessed from the beginning of cultivation to the last day without loss or damage to cells. However, many fundamental questions regarding the regulation and development of an hPSC colony as a multicellular population, including the relationship between morphological and pluripotent characteristics, and the clonal development of colonies, need further investigations.

In this study, we extracted and quantitatively analyzed seven morphological parameters of cells and colonies from three hPSC lines and linked these data to their clonality, pluripotency status, and differentiation ability towards three germ layers. We identified specific morphological parameters as the most informative ones in terms of variance between lines and different morphological features and used these parameters to train classification models of colony phenotypes. Finally, we linked the data of the morphological phenotype to the pluripotency status, reflecting the expression of pluripotency marker genes and differentiation markers, and further linked it to create the morphological “portrait” of the colony with a good phenotype. Our results indicated the fundamental possibility of constructing a morphological “portrait” of a colony informative for the automatic identification of the phenotype and link this portrait to the expression of pluripotency genes.

## 2. Results

**Figure 1:**
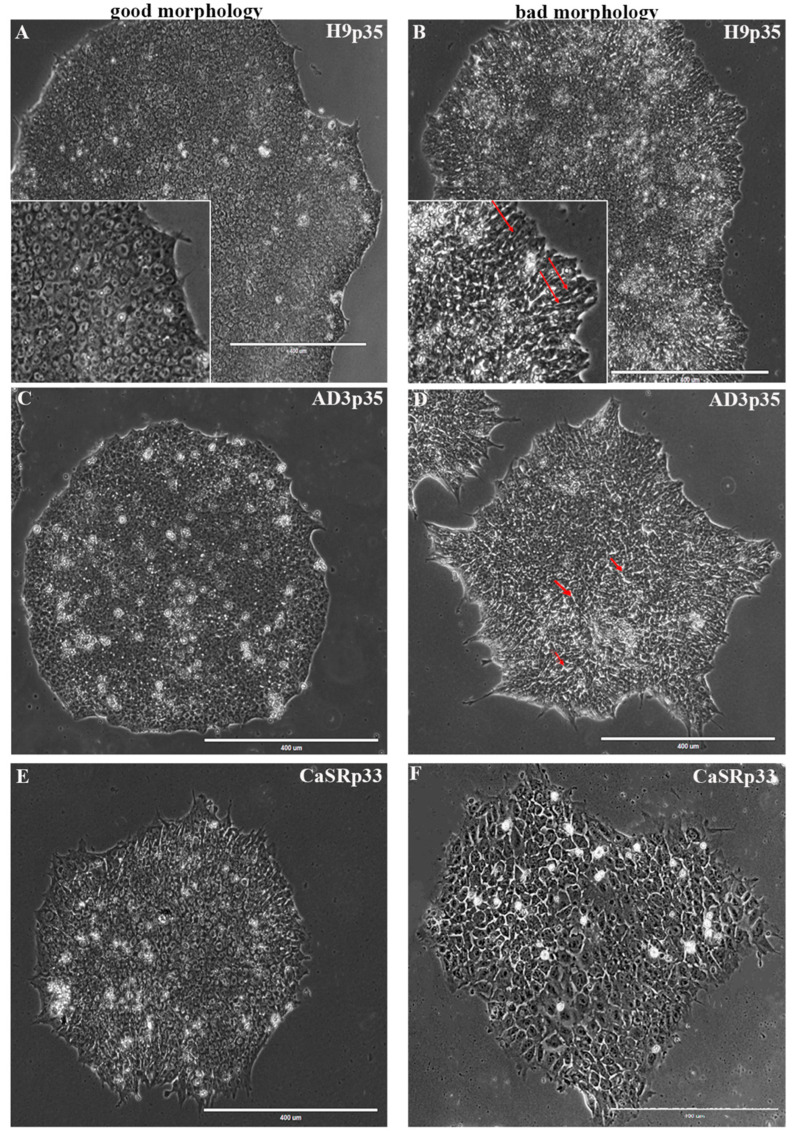
Morphological features of hPSC colonies with good and bad phenotypes at 120 h after plating. Phase-contrast observation. The left panels (**A**,**C**,**E**) show typical examples of hPSC colonies of good phenotype without signs of spontaneous differentiation: flat structure, prominent well–defined edge, and a high nuclear-to-cytoplasmic ratio, with prominent nucleoli in square-shaped, tightly packed cells. The right panels (**B**,**D**,**F**) show the typical morphology of colonies with bad appearance: altered cell morphology (long arrows indicate elongated cells) and loosely packed cells with phase-bright gaps visible between cells (small arrows). In addition to the lack of dense packing, note the “spiky” colony edge (**D**). For up to 3–4 days after plating, colonies may exhibit looser packing as the colonies spread out and become established. At this point, “spiky” colony edges should not be a cause for concern. The density of the colonies increases rapidly after 72–96 h, and the morphology changes significantly at 120 h, before the next passage. The cell sizes vary greatly (**F**), indicating the beginning of the spontaneous differentiation. Scale bar, 400 μm.

The routine culture of hPSCs presents some unique challenges when compared with culturing other human cell types. It is well accepted that healthy and high-quality hPSCs should exhibit the following characteristics: relatively round shapes; tightly packed cells with a high nucleus: cytoplasm ratio, where the nucleus practically inhabits the entire cell; prominent nucleoli; colony centers becoming very dense; and a well-defined edge (Figure 1).

**Figure 2:**
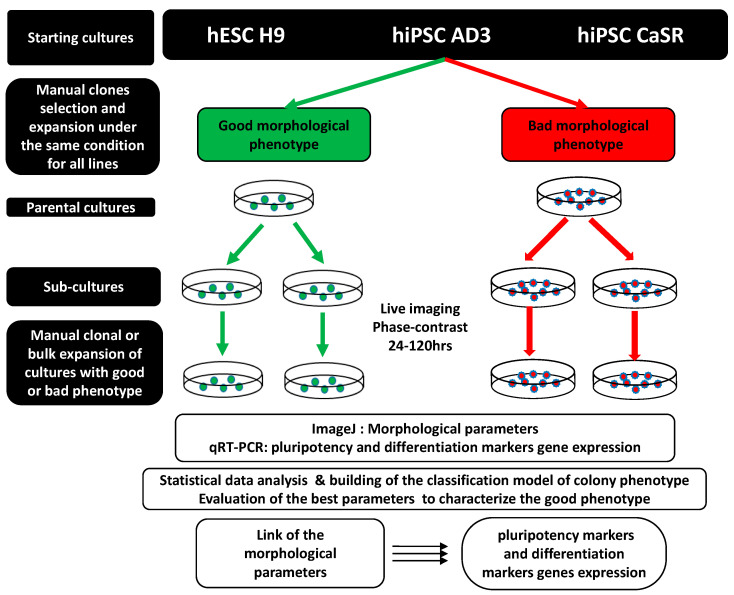
Schematic representation of the experimental design. Schematic representation of the experimental regime, in which hESC line H9 and two hiPSC lines (AD3 and CaSR) were cultured for an extended period under the same culture conditions. Stock cultures of all three lines were propagated manually by separately selecting cultures with good and bad colony morphology, and phase-contrast images were recorded for colonies of different morphological phenotype from 24 to 120 h in the early, middle, and late passages. Each colony of good or bad phenotype was then recloned manually to generate clonal subcultures. Parent cultures propagated via bulk expansion were further cultured under bulk conditions to generate nonclonal cohorts. At the end of the expansion period, cultures were propagated again manually or bulk-expanded to obtain cultures of good and bad morphological phenotypes from which morphological parameters (phase-contrast/ImageJ) and genetic material were extracted for gene expression via qRT-PCR analyses.

We extracted and analyzed seven parameters (Table 1) characterizing the morphology of growing colonies and cells from the phase-contrast images of hESC line H9, control hiPSC line AD3, and patient-specific hiPSC line CaSR in various passages during their growth under identical culture conditions for 120 h. The detailed schematic representation of the experimental design is presented in Figure 2.

Parameters Area and Perimeter describe the size of the cell’s/colony’s interior and boundary, respectively, while the Minor axis and Feret’s diameters represent the level of elongation. Shape factor quantifies the circularity of cells/colonies, thus representing a measure of their compactness. This parameter, also known as isoperimetric quotient, varies between 0 and 1, where 1 corresponds to the circularity of a circle and smaller values are associated with less circular and compact shapes. One of the indicators of the good quality of hPSCs is the ability for compactness as the colony reaches 72 h of culture. We quantified the compact packing of cells within colonies via the area of the free intercellular space (AIS), with larger values of this parameter being associated with less compact packing. Therefore, AIS represents an additional measure of compactness for colonies.

### 2.1. Variability in Morphological Parameters across Cell Lines and Growth Times

**Figure 3:**
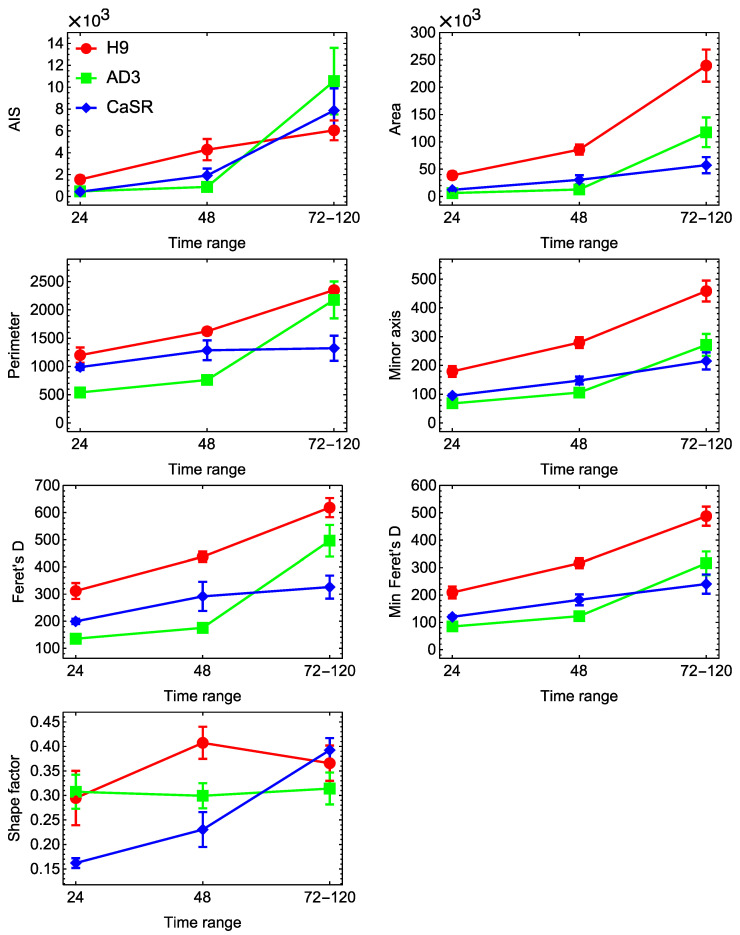
Dynamics of morphological parameters for colonies from three cell lines. The values of seven parameters from Table 1 were measured for all colonies from H9 (red), hiPSC AD3 (green), and hiPSC CaSR (blue) lines and all passages, at 24 h, 48 h, and 72–120 h. Mean values across processed colonies at each time point are shown, with the standard errors of the mean (SEMs) as the error bars. Late time points are grouped into one range. Shape factor, dimensionless parameter; AIS and Area, µm^2^; all other parameters, µm.

At first, we investigated how the morphological parameters of cells and colonies varied across lines, passages, and growing times. The colony growth process led to a gradual increase over time in most parameters, except Shape factor (Figure 3). The H9 colonies at all times generally demonstrated higher values of these parameters than the hiPSC lines, while the latter showed very close values. The dynamics of Shape factor were not monotonous and exhibited a qualitative difference between the H9 and hiPSC lines. Shape factor of H9 colonies reached a maximum at 48 h, meaning that the colonies became more regular in form by this time. This parameter stayed almost constant for hiPSC line AD3 and consistently increased for hiPSC line CaSR.

The analysis also showed that the variance in the morphological parameters of colonies from the same cell line came mostly from the time point changes rather than from the difference between various passages. For example, the comparison of control hiPSC colonies taken from the middle passage range (passages 21–28) and the late one (passages 39 and 56), fixed at the same time range (24–48 h) for each passage, did not show a significant difference (*p* > 0.05) in any parameter. These data probably point to the possibility to access the quality of the culture at the early time points (24–48 h) and to terminate it in the case of the sign of spontaneous differentiation.

**Figure 4:**
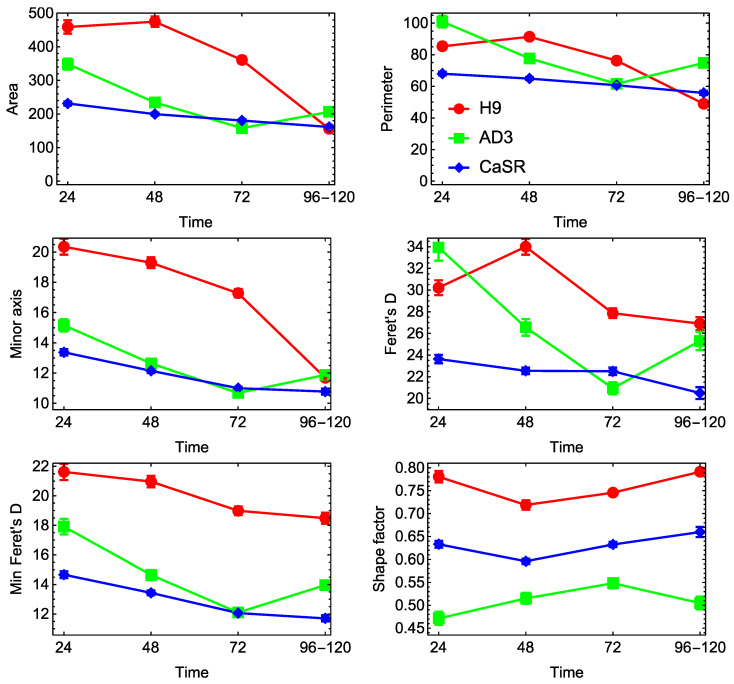
Dynamics of morphological parameters for cells from three cell lines. All designations are as in Figure 3.

The average morphological parameters of cells showed less consistent dynamics across lines and growing times (Figure 4). Most parameters of H9 cells decreased with time, which meant that the cells eventually became smaller as the colony became more tightly packed. A similar tendency was observed for hiPSC line CaSR, with the evident difference that cells from this line had a smaller size at the early and middle time points and showed less variance over time in all parameters. The parameters of cells from control hiPSC line AD3 demonstrated non-monotonous dynamics. The three lines were clearly and consistently separated from each other in terms of Shape factor, with H9 cells being the most regular in form and control hiPSCs being the least regular at all times (Figure 4). In contrast to colonies, differences in both the time point and passage made a notable contribution to the variance in cell parameters, as parameters of the same line and for the same growth time had statistically different mean values for different passages.

### 2.2. Variability in Morphological Parameters across Colony Phenotypes

A common practice in the process of hPSC colony propagation is the selection of healthy cultures for further expansion based on a visual analysis of their morphology related to the experience of specialists. Herein, we performed analyses of each colony from all lines based on their morphological appearance; thus, we defined the colony phenotype (“good” or “bad”) as a control parameter (Figure 1).

**Figure 5:**
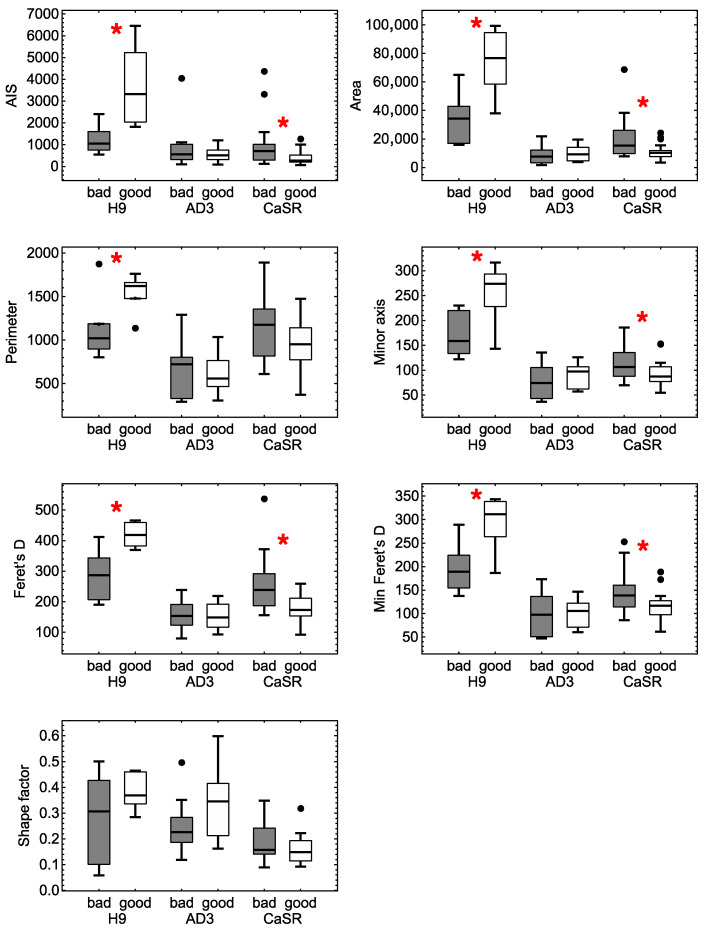
Boxplots showing the variance in colony morphological parameters within groups of different phenotypes for each cell line. Data comprise colonies in all passages and for growth times in the range of 24–48 h. Dots mark outliers. Asterisks indicate groups with a statistically significant (*p* < 0.05) difference in the mean values of the parameters.

To understand which colony morphological parameters were the most informative ones in relation to the phenotype, we analyzed the variance in these parameters in groups of different phenotypes and lines. The two-way analysis of variance showed that the interaction between the line and phenotype factors was statistically significant for all parameters (*p* < 0.05). A closer comparison of the groups demonstrated that different lines were characterized by different morphological parameters sensitive to phenotypic separation (Figure 5). For H9, colony parameters AIS, Area, Perimeter, Minor axis and both Feret’s diameters showed a significant difference (*p* < 0.05) in mean values between colonies with “bad” and “good” phenotypes. The same was true for hiPSC line CaSR, except for the fact that Perimeter only showed a trend towards statistical significance (*p* = 0.07). Therefore, these two lines exhibited similar sets of colony morphological parameters that could be considered as informative for assessing colony phenotypes. On the other hand, no colony parameter for hiPSC line AD3 showed a significant difference in mean values for different phenotypes (Figure 5). This meant that a single morphological characteristic of colonies was not enough to assess the phenotype of this line (even on average), so only their combination (a morphological portrait) should be tried for this purpose.

Interestingly, the results showed that the parameters of the hESC H9 line and hiPSC line CaSR associated with colony size appeared to be consistently associated with the phenotype, while of the two parameters characterizing compactness, only AIS was sensitive to the phenotype, thus emphasizing the importance of colony compactness as a reliable factor in the selection of the best quality clones. Shape factor showed visible variation among the groups, but that was not enough for statistical significance. This meant that colonies with different phenotypes in these lines had different internal cell packing, but different shapes could not be assessed at the level of this single parameter. We also saw a difference between the lines in the phenotype dependence on the average values of the relevant morphological parameters. These values increased for colonies with the good phenotype as compared with those with the bad phenotype for the H9 line, and this tendency was opposite for hiPSC line CaSR (Figure 5). This suggested that to identify various morphological phenotypes between different lines, it was necessary to turn to the development of the comprehensive analysis of the variability in various morphological parameters.

Next, we performed a similar analysis of the cellular morphological parameters (Figure S1). At the cellular level, the mean values of all parameters showed a statistically significant (*p* < 0.05) difference between the groups of good and bad phenotypes for hiPSC line AD3. Cells in hiPSC line AD3 colonies with the good phenotype on average had smaller mean values of Area, Perimeter, Minor axis, and both Feret’s diameters then cells with the bad phenotype, which could have been related to the more compact cells in good colonies. They were also more regular in form in the good colonies, which manifested as higher mean Shape factor for the good phenotype as compared with the bad one. Cells from the H9 line also demonstrated a statistically significant difference between phenotypes in the mean values of Shape factor, but in contrast to hiPSC line AD3, this change was associated with a lower average value of this parameter in the good colonies. This could probably indicate differences in colony growth patterns between these cell lines. We did not observe a statistically significant difference in the mean values of cell morphological parameters for hiPSC line CaSR (Figure S1).

### 2.3. Phenotype Classification Based on Morphological Parameters

As the variance analysis of single morphological parameters showed them to be informative for separating different phenotypes, we investigated the possibility to build a phenotype classification model using the morphological parameters as predictors. We combined the data of all cell lines into one data sample and first trained on this sample a classification model for predicting the phenotype based on all seven morphological parameters of colonies using neural networks (see Methods). The resulting cross-validation accuracy of this model was 70 ± 6%, indicating that the phenotype could be predicted with good precision when the parameters were considered in combination.

**Figure 6:**
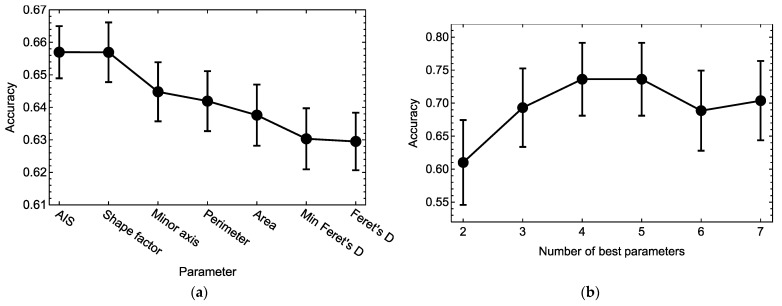
Mean accuracy ± SEM of the phenotype classification models based on different subsets of colony morphological parameters as predictors: (**a**) Importance measure for each parameter calculated as the mean cross-validation accuracy across the models containing this parameter in all possible combinations with all other parameters. Higher values of this measure for a parameter indicated that models containing this parameter as a predictor better recognized the colony phenotype. (**b**) Mean cross-validation accuracy of the models containing a given number of the best parameters from (**a**). Combinations of best parameters in (**b**): 2, AIS and Shape factor; 3, AIS, Shape factor and Minor axis; 4, AIS, Shape factor, Minor axis, and Perimeter; 5, AIS, Shape factor, Minor axis, Perimeter, and Area; 6, AIS, Shape factor, Minor axis, Perimeter, Area, and Min Feret’s D; 7, all parameters. The maximum accuracy in (**b**) was already achieved with four parameters, so the classification model with the four best parameters used as predictors provided a compromise between simplicity and prediction accuracy.

To find out which parameters were more informative in the context of such classification, we trained a set of classification models on the same data using all possible subsets of colony parameters as predictors, with the subset length ranging from two to seven parameters. As expected, the accuracy of these models grew almost monotonically as more parameters were added (Figure S2). We then calculated a measure of importance for each parameter by averaging the accuracy of all models containing this parameter. Higher values of this measure for a given parameter meant that the presence of this parameter in a model, on average, led to a higher accuracy of phenotype prediction in combination with any other morphological parameters. Sorting the parameters according to this importance measure demonstrated that AIS and Shape factor were the most informative for predicting the phenotype, and the mean accuracy of phenotype prediction of the models containing any of these parameters reached about 66% (Figure 6a). The presence of other parameters in the models led to lower accuracy values on average.

We combined a given number of the best parameters from Figure 6a, in decreasing order of their importance measure, and used each such combination of parameters as the only predictor in the phenotype classification model. Training these models on the data showed that the cross-validation accuracy of phenotype prediction as a function of the number of included best parameters approximately flattened out at four parameters (Figure 6b). The classification model with this number of best parameters (AIS, Shape factor, Minor axis, and Perimeter) gained a cross-validation accuracy of 74 ± 6%, and adding other parameters in the model did not improve this performance and could even make it worse (probably due to a higher tendency towards overfitting for a higher number of parameters). Therefore, we may consider this classification model as the minimal one in predicting the colony phenotype and regard this combination of colony parameters as the most informative traits of the true morphological portrait associated with the phenotype.

Applying the minimal classification model to the whole data sample led to a phenotype prediction accuracy of 77% (Table 2). Bad and good colonies were predicted with 72% and 83% precision, respectively. The misclassified colonies were distributed across the lines as follows: 10 (19%) for H9, 8 (17%) for hiPSC line AD3, and 15 (33%) for hiPSC line CaSR.

Next, we performed a similar analysis of the phenotype classification models based on cellular morphological parameters as predictors. Preliminary calculations showed that Feret’s D and Minimal Feret’s D contributed to the model accuracy in a measure comparable to the contribution of Minor axis, so we excluded these two parameters from consideration to simplify model training. A trained classification model including all other cell parameters (“full model”) produced a cross-validation accuracy of phenotype prediction equal to 69 ± 3%, which was less accurate than when colonial parameters were used as predictors.

**Figure 7:**
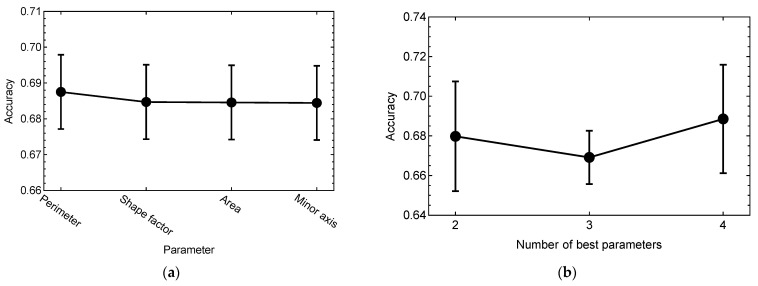
Same as in Figure 6 but for classification models based on the four cellular morphological parameters. (**a**) Importance measure for each parameter. (**b**) Mean cross-validation accuracy of the models containing a given number of the best parameters from (**a**). Combinations of best parameters in (**b**): 2, Perimeter and Shape factor; 3, Perimeter, Shape factor, and Area; 4, Perimeter, Shape factor, Area, and Minor axis.

Classification models based on all possible subsets of the four cellular parameters showed that on average, three parameters were sufficient to reach the highest possible accuracy (Figure S3). The analysis of these models revealed that all parameters demonstrated approximately the same contribution, with a slightly larger contribution from Perimeter (Figure 7a). Only two of the best parameters from Figure 7a (Perimeter and Shape factor) were sufficient to train the classification model with an accuracy of 68 ± 3%, which was close to the observed maximal accuracy of 69% (Figure 7b). Therefore, we could consider the classification model including these two parameters as the minimal model for colony phenotype prediction based on the cellular data.

Applying the minimal model based on the cellular parameters to the whole data sample led to a phenotype prediction accuracy of 69% (Table 3). Bad and good colonies were predicted with the 66% and 71% precision, respectively. The misclassified cells were distributed across the lines as follows: 130 (11%) for H9, 195 (39%) for hiPSC line AD3, and 569 (48%) for hiPSC line CaSR. Due to the high numbers of misclassified cells for the hiPSC lines, we trained the classification models on the four cellular parameters separately for each line and obtained classification accuracy values close to the model based on all-lines data: 71 ± 12% (H9), 65 ± 8% (hiPSC line AD3), and 66 ± 5% (hiPSC line CaSR).

### 2.4. Expression of Pluripotent and Differentiation Marker Genes

**Figure 8:**
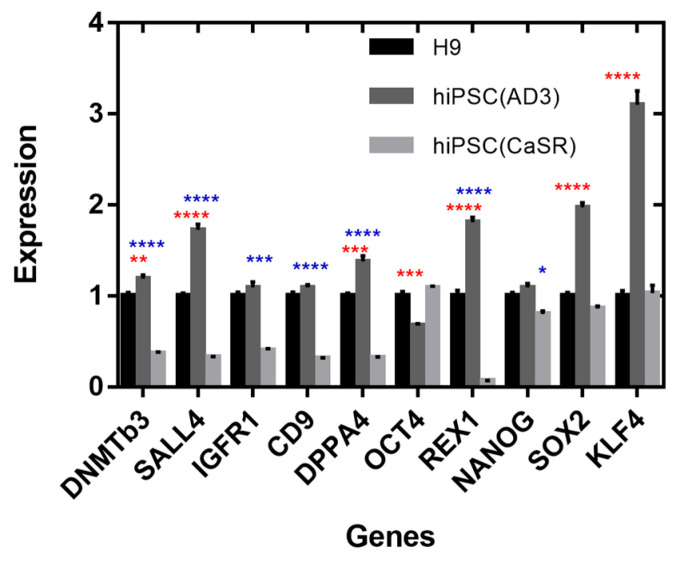
Relative expression of ten pluripotent genes measured in three cell lines using qRT-PCR. Data are presented as means ± SEMs. Values were normalized to the mean expression in the H9 line. Red asterisks point out the comparison of gene expression between hESC line H9 and hiPSC line AD3, and blue asterisks point out the comparison between hESC line H9 and hiPSC line CaSR. * *p* < 0.05; ** *p* < 0.01; *** *p* < 0.001; **** *p* < 0.0001.

To reveal how the expression of pluripotency markers depended on the clonality and morphological types of hPSCs, we measured the expression of ten important pluripotent markers (*DNMTB3*, *SALL4*, *IGFR1*, *CD9*, *DPPA4*, *OCT4*, *REX1*, *NANOG*, *SOX2*, and *KLF4* genes) in colonies from the three cell lines with different clonality statuses and phenotypes. By comparing the quantitative gene expression levels between all cell lines, we found that hiPSC line AD3 was mostly characterized by an elevated expression as compared with H9, while some markers that differentiated hiPSC line CaSR from H9 tended to be underexpressed in hiPSC line CaSR (Figure 8). The most prominently overexpressed genes in hiPSC line AD3 compared with H9 were (in descending order of apparent effect) *KLF4*, *SOX2*, *REX1*, *SALL4*, *DPPA4*, and *DNMTB3*, while *OCT4* showed underexpression. For hiPSC line CaSR, the most underexpressed genes compared with H9 included *REX1*, *SALL4*, *CD9*, *DPPA4*, *DNMTB3*, *IGFR1*, and *NANOG*, with no overexpressed genes.

**Figure 9:**
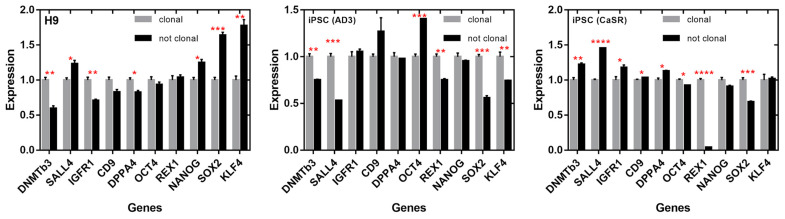
Relative expression of ten pluripotent genes in clonal (grey) and nonclonal (black) colonies measured for each cell line using qRT-PCR. Data are shown as means ± SEMs. Values were normalized to the mean expression in clonal colonies separately for each line. * *p* < 0.05; ** *p* < 0.01; *** *p* < 0.001; **** *p* < 0.0001.

To further investigate a possibility for pluripotent markers to differentiate between colonies with different clonality statuses, we plotted the expression in clonal and nonclonal colonies for each cell line (Figure 9). Nonclonal colonies exhibited underexpression of *REX1* (in both hiPSC lines), *SALL4* (hiPSC line AD3), *SOX2* (both hiPSC lines), *DNMTB3* (in H9 and hiPSC line AD3), *IGFR1* (H9), *KLF4* (hiPSC line AD3), *DPPA4* (H9), and *OCT4* (hiPSC line CaSR). The following genes were overexpressed in nonclonal colonies compared with clonal ones: *SOX2* (H9), *OCT4* (hiPSC line AD3), *SALL4* (H9 and hiPSC line CaSR), *KLF4* (H9), *DNMTB3* (hiPSC line CaSR), *NANOG* (H9), *IGFR1* (hiPSC line CaSR), *CD9* (hiPSC line CaSR), and *DPPA4* (hiPSC line CaSR). The fact that the sign of change in expression (over- or underexpression) of many genes varied across the lines pointed to the existence of line-dependent mechanisms that control pluripotency, in agreement with previous publications [18,19].

**Figure 10:**
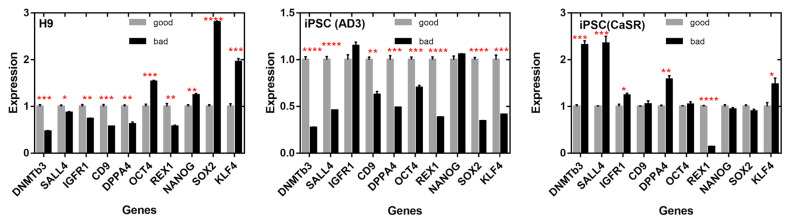
Relative expression of ten pluripotent genes in “bad” (black) and “good” (grey) colonies, according to morphological phenotype, measured for each cell line using qRT-PCR. Data are presented as means ± SEMs. Values were normalized to the mean expression in good colonies separately for each line. * *p* < 0.05; ** *p* < 0.01; *** *p* < 0.001; **** *p* < 0.0001.

We performed a similar analysis relating gene expression to the colony phenotype (Figure 10). In hiPSC line AD3, bad colonies were differentiated from the good ones by the essential underexpression of all pluripotent markers except for *IGFR1* and *NANOG*, which showed no significant variations between phenotypes. In H9, four genes (*SOX2*, *KLF4*, *OCT4*, and *NANOG*) were overexpressed, and all the others were underexpressed in colonies with bad morphology. In hiPSC line CaSR, bad colonies were characterized by the overexpression of *DNMTB3*, *SALL4*, *DPPA4*, *KLF4*, and *IGFR1* and by the underexpression of *REX1*. Therefore, a change in clonality or phenotype could be associated, in a line-dependent manner, with a change in the expression of the mentioned pluripotent markers.

Since one of the important characteristics of hPSCs is their ability to differentiate into three germ layers, we first addressed using immunofluorescence the differentiation abilities of embryonic bodies (EBs) formed from hESC line H9 and hiPSC line AD3 with the clonal or bulk expansion of PSCs. In all cases, cells were able to differentiate into three germ layers, as confirmed with the positive staining of ectodermal lineage (TUJ1), mesodermal lineage (SMA), and endodermal lineage (AFP) (Figure S4). The next question we asked was the following: Is there a link among the morphological phenotype, the clonal vs. bulk expansion of the line, and the expression of the differentiation markers during EB development? In other words, how sensitive is the differentiation marker expression to the phenotype of the cell line from which EBs are generated? For this purpose, we measured the expression of ten differentiation marker genes *(NESTIN*, *SOX1, MSX2*, *CDX2*, *HAND1*, *RUNX2*, *MIXL1*, *BRACHYURY*(*T*), *SOX17*, and *GATA4*) along with *DPPA4*, *OCT4*, and *NANOG* in differentiating EBs derived from the H9 line and the hiPSC AD3 line, with each gene expression being studied in two cultures (clonal and nonclonal) of each line (Figure S5). Despite many of these genes exhibited visual difference in expression between the EBs originated from clonal and nonclonal colonies of H9, only *HAND1* demonstrated a statistically significant (*p* < 0.05) overexpression in the EBs grown from cells of clonal origin. In contrast, multiple markers significantly differentiated EBs from clonal colonies from nonclonal ones of the hiPSC AD3 line; *RUNX2* and *CDX2* were overexpressed, and *NANOG*, *BRACHYURY*(*T*), and *SOX17* were underexpressed in the EBs grown from clonal colonies compared with those originated from nonclonal ones. Several genes showed significantly different expression between the lines. The EBs from nonclonal colonies of H9 were differentiated from the EBs of nonclonal colonies of hiPSC line AD3 by the underexpression of *NANOG*, *DPPA4*, *BRACHYURY*(*T*), and *SOX17* and by the overexpression of *OCT4*. The EBs from clonal colonies of H9 were differentiated from the EBs formed from clonal colonies of hiPSC line AD3 by the underexpression of *CDX2* and *GATA4* and by the overexpression of *MSX2* (Figure S5). The revealed expression variations in the EBs originated from two different hPSC lines propagated under strict manual (clonal) or bulk (nonclonal) expansion may have been associated with differentiation bias and highlighted various differentiation propensity of EBs of various origins, in good agreement with previous data [19].

## 3. Discussion

Human pluripotent stem cells demonstrate high cellular variability, and their utility is further limited by the cellular phenotypic changes that are frequently observed following prolonged culture [7,20]. Therefore, the routine characterization of hPSCs using several standard criteria, such as cell growth, marker expression, karyotype analysis, and in vitro differentiation, is required to confirm hPSC status and viability [21]. Colony morphology is one such criterion that is used to continuously evaluate hPSC health. Typical healthy undifferentiated hPSCs appear as tightly packed, round or squared cells with large nuclei and notable nucleoli without spaces between cells [22,23]. The morphology of differentiated and unhealthy hPSCs differs from that of normal ones, and several studies already showed that the morphology of colonies correlates with their quality [16,24,25].

Human pluripotent stem cells are usually clonally derived. Generally, the selection of fully reprogrammed cells involves the picking of individual colonies with good morphology similar to that of hESCs. The selection of the best colonies is, however, difficult to standardize, as it is rather based on the experience of the researcher than on objective criterium [8]. Moreover, different hiPSC lines show considerable phenotypic variations, and the lack of common well-characterized morphological parameters frustrates efforts to integrate data across research groups or replicate some findings. Therefore, this procedure needs to be more standardized.

Until now, the parametric characteristics of morphological characters associated with the pluripotent status of hPSCs were insufficiently studied. Herein, we present a systematic approach to this problem by quantifying a set of morphological parameters of colonies and cells of three human pluripotent cell lines, phenotyping colonies according to their potential pluripotency, and linking these types of information using statistical and machine learning methods. Specifically, we extracted and characterized seven morphological parameters and demonstrated that AIS and Shape factor were the most informative ones for the predicting and recognizing the good phenotype of hPSCs. Our results showed that the colonial and cellular morphological parameters had different variability patterns across cell lines and growth times, thus suggesting that these were two complementary types of information about the true morphological portrait of the colony. These parameters also exhibited different variance patterns across colony phenotypes, but both colonial (AIS and Shape factor) and cellular individual parameters (Feret’s D, Minor Axis, and Perimeter) were capable, on average, of distinguishing colonies with good and bad phenotypes.

Based on the phenotype recognition of the culture, we further tested the ability of its morphological portrait to be linked with pluripotent and differentiation gene expression. The analysis of pluripotent marker expression in the genetically three different cell lines supported the validity of our approach. The ability of the *SALL4, DNMTB3, REX1, DPPA4*, and *SOX2* genes to distinguish well between different phenotypes of hPSC colonies implied that the variable phenotypes we used in the study did represent the pluripotency status of the colony. The expression of multiple genes was also a good factor for separating colonies with different clonality, both in the cell lines and in the EBs derived from them. Recently, a work by Bjørlykke and colleagues reported that reprogrammed cells displayed distinct proteomic signatures associated with the variability in colony morphology in 20 hiPSC lines [26]. The proteome data analysis presented in this study showed that different morphological portraits of colonies were associated with different proteomic profiles and different competencies for directed differentiation. For the first time, the presence of a relationship between pluripotent markers (*DNMT3B, DPPA4, SALL4,* and *CD9*) and morphological portraits of various lineages was shown [26], thus supporting our data. In addition, and in good agreement with previous data [26], two master pluripotency transcription factors, namely, *OCT4* and *NANOG*, showed poor ability to distinguish between colonies with good and bad morphological phenotypes, suggesting the importance to include in the hPSC score list additional genes, such as *SALL4, DNMTB3, REX1*, and *DPPA4.*

Next, we presented classification models that were able to predict colony phenotypes with a good precision based on the values of morphological parameters. A few relevant parameters were enough to reproduce this precision. Among the colony parameters, AIS and Shape factor turned out to be the most informative ones for phenotype classification. They estimated the colony form (its circularity) and its internal compactness, respectively. Interestingly, Shape factor was not identified as significant in the variance analysis, suggesting that the interaction between several parameters is important in a good classification model. Among the cellular parameters, Perimeter and Shape factor were the most informative ones. This difference between the colonial and cellular parameters could be considered as another manifestation of different phenotypic information encoded at the level of cells and the whole colony.

The underlying reasons for the dynamic change in hPSC colony morphology are not fully understood. Among them, epigenetic memory, incomplete reprogramming, the feeder-free system, and the competition of elite cells could be some of the reasons [27,28,29]. In addition, the variation in hiPSC lines was shown to be donor dependent [30,31]. However, variations related to colony morphology are not donor dependent [26], thus highlighting the urgent need to deepen our knowledge about the laws that regulate hPSC morphology.

Without doubt, our study did not provide a complete picture. The diversity of colony morphology should be expanded, and the correlation between colony morphology and gene expression should be evaluated using other hPSC lines and bioimaging informatic analyses [32,33]. There is also room for improvement in the performance of our phenotype classification models, as their average classification accuracy was below the possible maximum. This could be performed by expanding the training data, which would allow the development of models specific to different conditions (different lines, passages, and growing times) to be performed. The classification models in our study were trained on the data combined from the three cell lines, so these models learned rather general features separating the bad and good phenotypes of different colonies in different cell lines. In particular, these general models demonstrated lower performance on hiPSC line CaSR data. Models trained on data specific to a cell line are important, as they should be more sensitive to line-specific features. With larger data sets, more sophisticated machine learning methods could also be applied.

**Figure 11:**
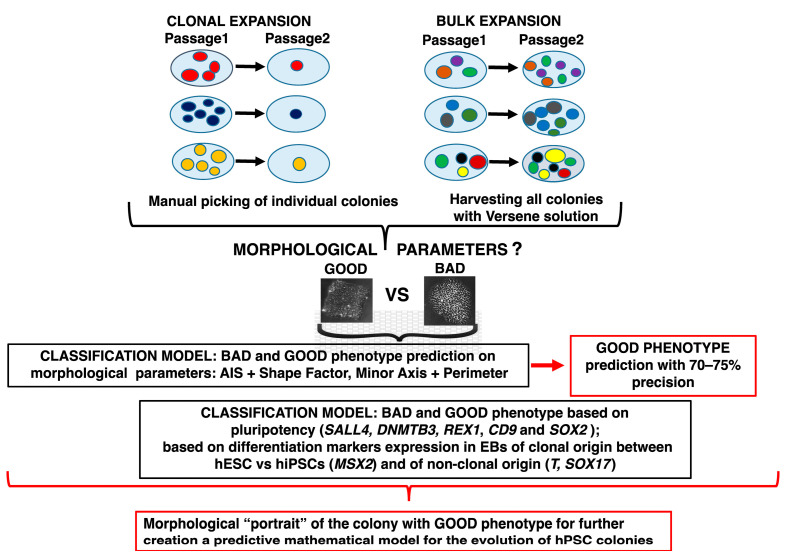
Summary of results. Our paper describes the mathematical classification model built on the morphological parameters of hPSC lines according to their morphological phenotypes and clonal vs. nonclonal (bulk) expansion. The most informative parameters were AIS and Shape factor (for colonies), and Minor axis and Perimeter (for cells). In the next step, we linked pluripotency and differentiation factor expression to the morphological phenotype and to the type of the culture expansion (clonal vs. nonclonal/bulk), revealing the importance of *SALL4*, *DNMTB3*, *REX1*, *CD9*, and *SOX2* pluripotency markers for the recognition of the colony phenotype, while differentiation marker *MSX2* was sensitive for discriminating the clonal origin of the cells. *T* and *SOX17* were informative for distinguishing the nonclonal EB origin. The models demonstrated the possibility of constructing the morphological “portrait” of a colony informative for the automatic identification of the phenotype and for linking this portrait to the expression of pluripotency genes.

We summarized our results in Figure 11. Overall, these results demonstrated the ability to assess the true morphological portrait of a colony that reflected its pluripotency and clonality properties. We identified specific informative parameters for that aim and built phenotype classification models, which can be used in further applications. The automation of cell culture and the high-throughput generation of hiPSCs would provide fascinating perspectives for drug screening and the analysis of patient-specific hiPSCs, along with their safe clinical application. These results can be considered as the first step towards constructing a predictive mathematical model for the evolution of human pluripotent stem cell colonies.

## 4. Materials and Methods

### 4.1. Cell Culture and Propagation

Human embryonic stem cell line H9 (WiCell, Madison, WI, USA), hiPSC line AD3 [5], and patient-specific hiPSC line HPCASRi002-A (CaSR) [6] were passaged on 6-well plates coated with hESC-qualified Matrigel Matrix (Corning Matrigel Matrix, Life Sciences) manually or via bulk expansion, at a 1:4 split ratio using 0.02% EDTA (Versene) dissociation solution and 10 µM ROCK inhibitor (Y-27632; StemCell Technologies). A volume of 2 mL of mTERSR1 medium (StemCell Technologies) was used per well daily for all cultures. All cell cultures were checked daily. During manual colony propagation, small cell clumps of 15–20 cells per clamp were used from the colony. All cultures were kept under standard condition for 5 days at 37 °C with 5% CO_2_ atmosphere and 21% O_2_ according to WiCell Inc. protocols. Phase-contrast imaging analyses of colonies were carried out 24, 48, 72, 96, and 120 h after plating before they reached 70% confluency across the well. For time-lapse recording, image capture was performed with CellVoyager CQ1 High-Content Analysis System (Yokogawa, Japan) every 5 h.

The morphological parameters of cells and colonies were extracted using the FlowJo [34] analysis of the phase-contrast images of all cultures in various passages (early (12–14 p), middle (21–28 p), and late passages (36–56)), during their growth for 120 h, as previously described [23]. In brief, cells and colonies were manually removed from the background using Adobe Photoshop software (v. 21.1.0.106, Adobe Inc., San Jose, CA, U.S.A.). The ImageJ program was used to calculate parameters such as Area, Perimeter, Minor axis, Feret’s diameter, minimal Feret’s diameter, Shape factor, and AIS (only for colonies) using a built-in particle analyzer.

The parameter values were obtained for 53 colonies and 1602 cells of hESC line H9, 49 colonies and 1569 cells of control hiPSC line AD3, and 48 colonies and 1315 cells of patient-specific hiPSC line CaSR.

### 4.2. In Vitro Trilineage Differentiation

To functionally examine the pluripotency of hPSC lines, their differentiation potential towards three germ layers was evaluated using embryoid body (EB) formation. In brief, undifferentiated colonies of all hPSC lines in middle passages (26–28 p) were enzymatically detached (Versene solution; Paneco) and replated in ultra-low attachment plates (Corning, Corning, NY, USA) with mTeSR™-1 medium (StemCell Technologies, Vancouver, BC, Canada) and Rock inhibitor (StemCell Technologies). The next day, cells that formed aggregates were transferred to a new ultra-low attachment plate to form EBs under differentiation medium DMEM-F12 (Invitrogen, Waltham, MA, USA), 20% (*v*/*v*) Knockout Serum Replacement (Invitrogen), 1% L-glutamine, 1% nonessential amino acids (NEAA; Invitrogen), and 1% penicillin-streptomycin (Invitrogen). After 7 days of EB cultures in suspension, EBs were transferred to a 12-well plate covered with 0.1% gelatin and were allowed to attach for further differentiation over 10 days. On day 10, the differentiated EBs were stained for germ layer markers with antibodies anti-TUJ1 (BioLegend, San Diego, CA, USA; 801213), anti-AFP (GeneTex, Irvine, CA, USA; GTX131311), and anti-alpha-SMA (Sigma Aldrich, St. Louis, MO, USA; A2547-.2ML), or cells were harvested for RNA isolation.

### 4.3. Quantitative RT-PCR

To analyze gene expression, total RNA was isolated with Aurum™ Total RNA Mini Kit (BioRad, Hercules, CA, USA) according to the manufacturer’s instructions. RNA was quantified using NanoDrop ND-1000 Spectrophotometer (NanoDrop Technologies, Inc, Wilmington, DE, USA). cDNA was obtained via the reverse transcription of RNA using RevertAid H Minus First Strand cDNA Synthesis Kit (Thermo Fisher Scientific, Lithuania) according to the manufacturer’s instructions. For qRT-PCR, cDNA was amplified with specific primers, using qPCRmix-HS SYBR (Evrogen, Moscow, Russia) in the BioRad CFX Opus-96 real-time system (BioRad, Hercules, CA, USA) according to the kit’s enclosed protocol. The expression of target genes was normalized to the *18S* gene. Primers are presented in Table S1. All amplifications were performed in triplicates. All experiments were performed as three biological repeats. The statistical analysis of expression data was performed using GraphPad Prism software, version 7.0 (San Diego, CA, U.S.A.). We investigated the panel of 3 housekeeping genes: *RN18S*, *GAPDH*, and *RPLI3A*. *RN18S* had the best stability in all cases; the expression of target genes was normalized to *RN18S*.

### 4.4. Statistical Analysis and Classification Model

The comparison of the means in the statistical analysis was performed by applying either the *t*-test or the Mann–Whitney test, depending on the properties of data samples. The classification models were trained and validated using function *Classify* implemented in Wolfram Mathematica software [35]. This function provides an automated classification approach by selecting the best configuration (classification method along with its hyperparameters) of the classifier, similar to the Hyperband method [36]. The function tries to find the best configuration by training multiple models with random configurations on smaller data subsets and testing their performance on the full data set. As a measure of classification quality, the function produces a cross-validation estimate (with standard error) for classification accuracy. In most of our computational experiments, we found that the resulted accuracy was higher if we fixed the neural network as the classification method. We used the following command for a typical run of the training process in a Mathematica notebook: *Classify*[data, *Method* → “*NeuralNetwork*”, *ValidationSet* → *Automatic*, *PerformanceGoal* → “*Quality*”], where “data” is a list of data values in the form: list of parameter values for a colony/cell → phenotype. With this form of *ValidationSet* option, the validation set was selected automatically from “data” during the model training. As a control, we performed computational experiments on classification on the cellular data with the manual separation of the training, validation, and testing data sets and obtained the same model performance (Text S1, Figure S6). We did not apply any preprocessing to the data.

## 5. Conclusions

The observation of hPSC colony morphology before further propagation is an important step for their assessment. Practically, in research laboratories and stem cell banks, the best clones are selected based on their morphological appearance, but the data about the statistical evaluation of the morphological parameters between different lines and how they reflect the pluripotent status of the colony are very limited. This limitation further hinders the development and creation of new approaches for non-invasive methods for the best hPSC clone selection. We approached this limitation by generating a classification model based on informative morphological parameters as predictors of colonies with the good or bad phenotype from different hPSC lines. The most informative parameters, which allowed us to predict the good clones with 70–75% precision, were AIS and Shape factor (from the colony data), and Minor axis and Perimeter (from the cell data). The classification models demonstrated lower performance on hiPSC line CaSR data. To find out the link between pluripotency and clonality in different lines, we examined pluripotency marker expression in hPSC colonies with good and bad morphological phenotypes of clonal and nonclonal (bulk) expansion. We observed significant changes in the expression levels of the pluripotency marker genes in a line- and clonality-dependent manner. Among them, *DNMTB3*, *SALL4*, *REX1*, *CD9*, and *SOX2* were sensitive pluripotency markers to address the clonality and morphological phenotype of the lines. The expression of differentiation marker *MSX2* was a sensitive predictor of clonality, while *T* and *SOX17* were sensitive markers of EBs of nonclonal origin. We consider the phenotype classification models built in our study as a first step towards the creation of a mathematical model for the evolution of hPSC colonies.

## Figures and Tables

**Table 1:** Morphological parameters extracted from the images of cells and colonies.

Parameter	Description
Area	Area of the cell or colony
Perimeter	Length of the cell or colony boundary
Minor axis	Length of the minor axis of the ellipse fitted to the cell or colony
Feret’s diameter D	Largest distance between two points on the cell or colony boundary
Minimal Feret’s diameter D	Smallest distance between two points on the cell or colony boundary
Shape factor	Area divided by squared perimeter and multiplied by 4π (measure of circularity and compactness)
Area of intercellular space (AIS) ^1^	Total area of the free intercellular space in the colony (measure of compact cell packing)

^1^ This parameter is only for colonies.

**Table 2:** Confusion matrix for the minimal classification model based on colony parameters, showing the numbers of correctly and incorrectly classified colonies in the groups of the good and bad phenotypes. The first column shows (top) the number of correctly classified colonies with the bad phenotype, (middle) the number of good colonies misclassified as bad, and (bottom) the total number of colonies classified by the model as having the bad phenotype. The second column shows (top) the number of bad colonies misclassified as good, (middle) the number of correctly classified colonies with the good phenotype, and (bottom) the total number of colonies classified by the model as having the good phenotype. The third column shows the total number of (top) bad and (bottom) good colonies.

	Bad Phenotype, Predicted	Good Phenotype, Predicted	Total in the Class, Observed
**Bad phenotype**, **observed**	55	21	76
**Good phenotype**, **observed**	12	58	70
**Total in the class**, **predicted**	67	79	

**Table 3:** Confusion matrix for the minimal classification model based on cellular parameters, showing the numbers of correctly and incorrectly classified cells in the groups of the good and bad phenotypes of their colonies. The numbers have the same meaning as in Table 2 but refer to cells, not colonies.

	Bad Phenotype, Predicted	Good Phenotype, Predicted	Total in the Class, Observed
**Bad phenotype**, **observed**	720	379	1099
**Good phenotype**, **observed**	513	1248	1761
**Total in the class**, **predicted**	1233	1627	

## Data Availability

The data sets with measured colonial and cellular parameters, along with phenotype information, can be found at the Zenodo repository (https://doi.org/10.5281/zenodo.7150644 (accessed on 5 October 2022)).

## Supplementary Materials

The following supporting information can be downloaded at: https://www.mdpi.com/article/10.3390/ijms232112902/s1.
