# Supplementary material for: Prognostic Analysis of Human Pluripotent Stem Cells Based on Their Morphological Portrait and Expression of Pluripotent Markers
Source: Int J Mol Sci. 2022 Oct 26;23(21):12902. doi: 10.3390/ijms232112902 (PMC9656397; doi:10.3390/ijms232112902)
Supplement: Supplementary file 1 [file ijms-23-12902-s001.zip › ijms-1945337-supplementary.pdf]

Supporting Information

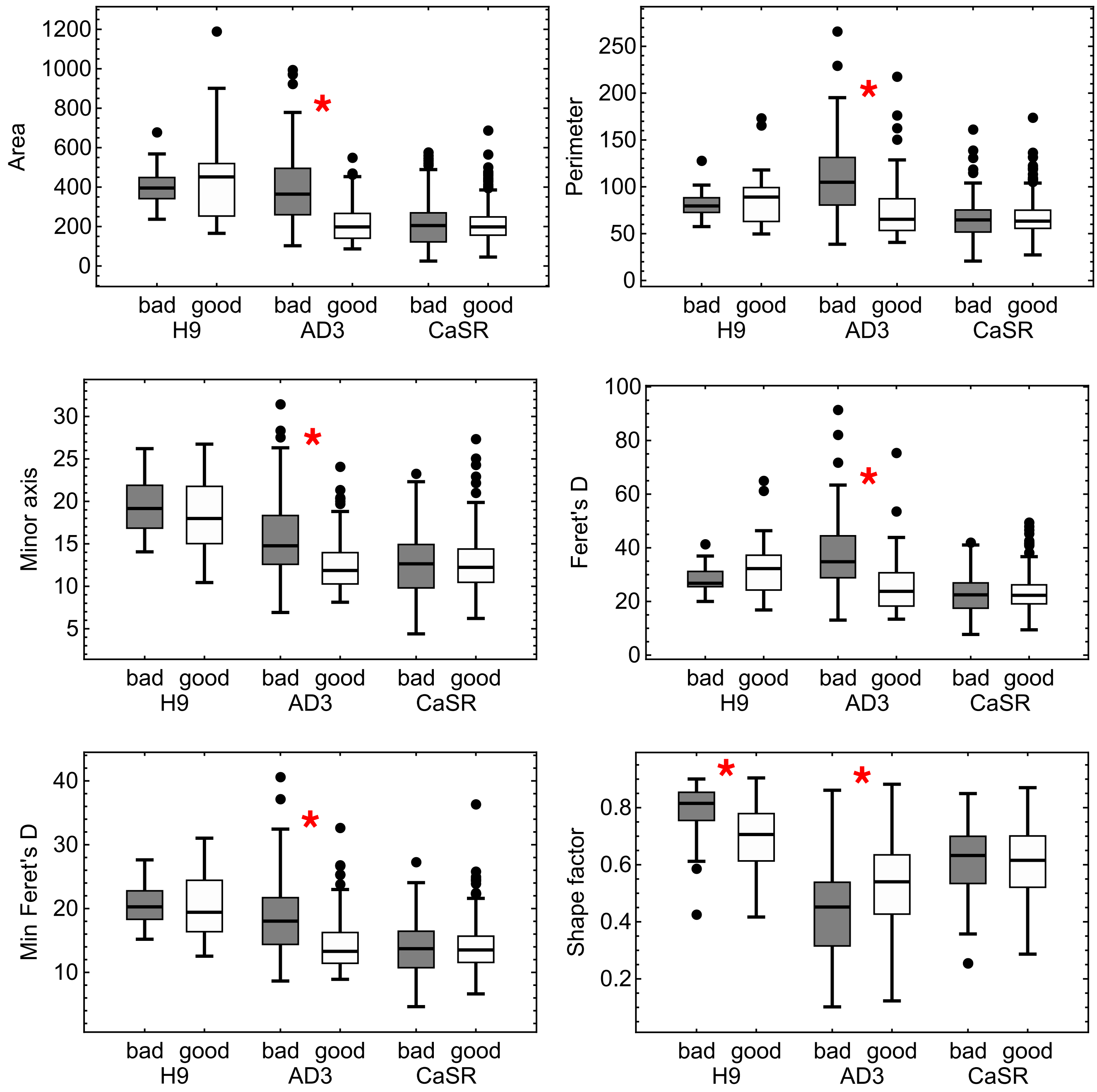

Figure S1. Boxplots showing the variance of cell morphological parameters within groups of different phenotypes for each cell line. Data comprises cells from all passages and for growth times in the range 24–48 hours. Dots mark outliers. Asterisks indicate groups with a statistically significant ($P < 0{,}05$) difference between the mean values of the parameters.

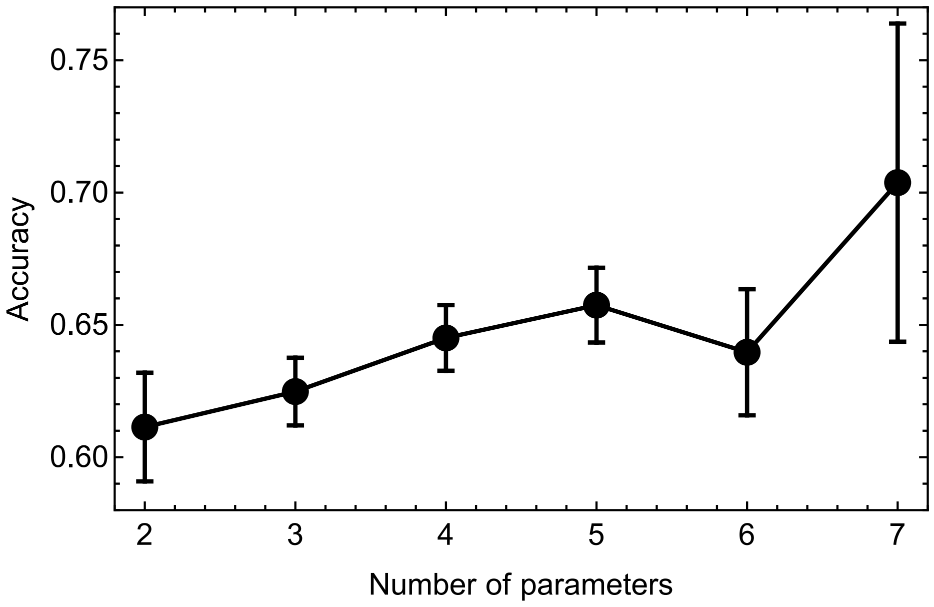

Figure S2. Mean accuracy of the phenotype classification models based on different subsets of colony morphological parameters as predictors. The values were obtained by averaging the cross-validation accuracies of classification models based on all possible subsets containing a given number of parameters (starting with two parameters). The error bars show the standard error of the mean.

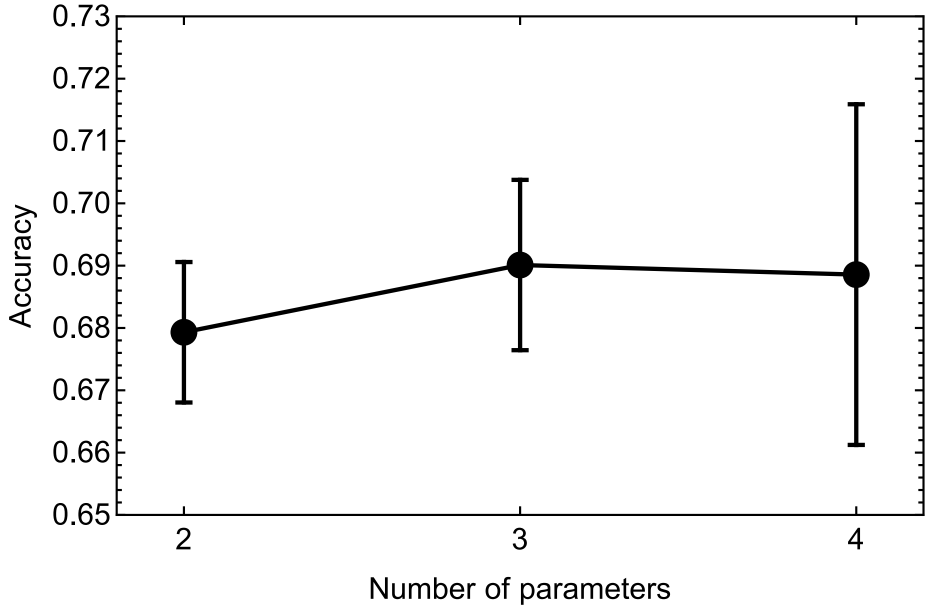

Figure S3. Mean accuracy of the phenotype classification models based on different subsets of four cell morphological parameters as predictors (Area, Perimeter, Minor axis, and Shape factor). The values were obtained by averaging the cross-validation accuracies of classification models based on all possible subsets containing a given number of cell parameters (starting with two parameters). The error bars show the standard error of the mean.

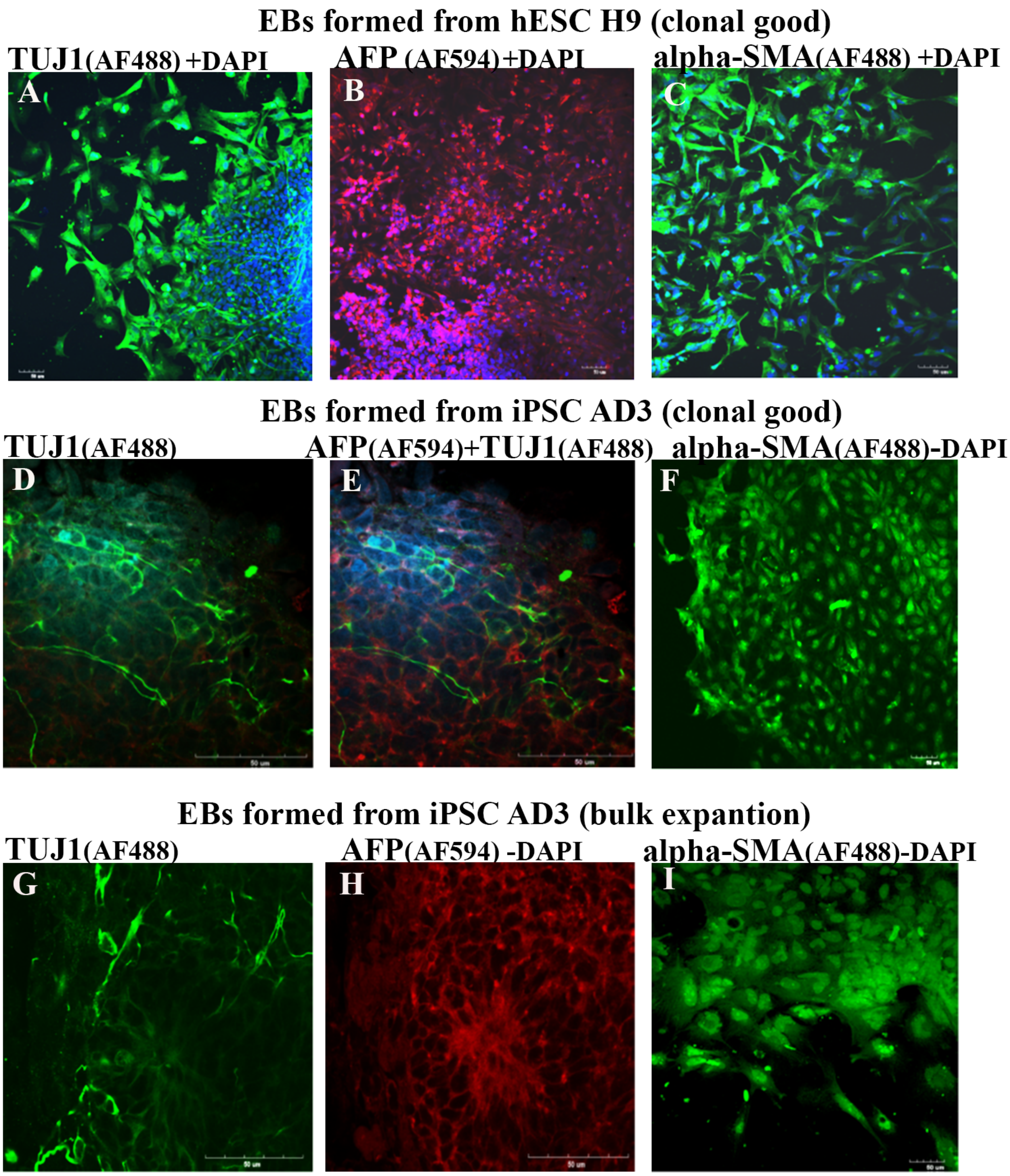

Figure S4. Fluorescence immunostaining of embryonic bodies (EBs) derived from hESC H9 and hiPSC AD3 after their manual clonal and bulk expansion. Representative positive confocal immunostaining images for common three germ layers markers: beta-III tubulin (TUJ1): for ectoderm, smooth muscle actin (alpha-SMA) for mesoderm, and alpha-fetoprotein (AFP) for endoderm. Immunofluorescence using this set of germ layer markers is particularly advantageous in that each of these markers is not only preferentially expressed in certain cell lineages, but each marker also displays a distinguishing cellular localization pattern that serves as an extra measure of visual confirmation. Please note an anti-TUJ1 staining is typically brightest for elongated cells with characteristic neuronal like projections (**A, D, G**). Cells that robustly express SMA display lined patterns of actin filament staining, which cells are often found in large layered groupings or as isolated, large cells (**C, F, I**). Anti-AFP staining commonly identifies tight clusters of cells (**B, E, H**). Scale bar: 50 μm.

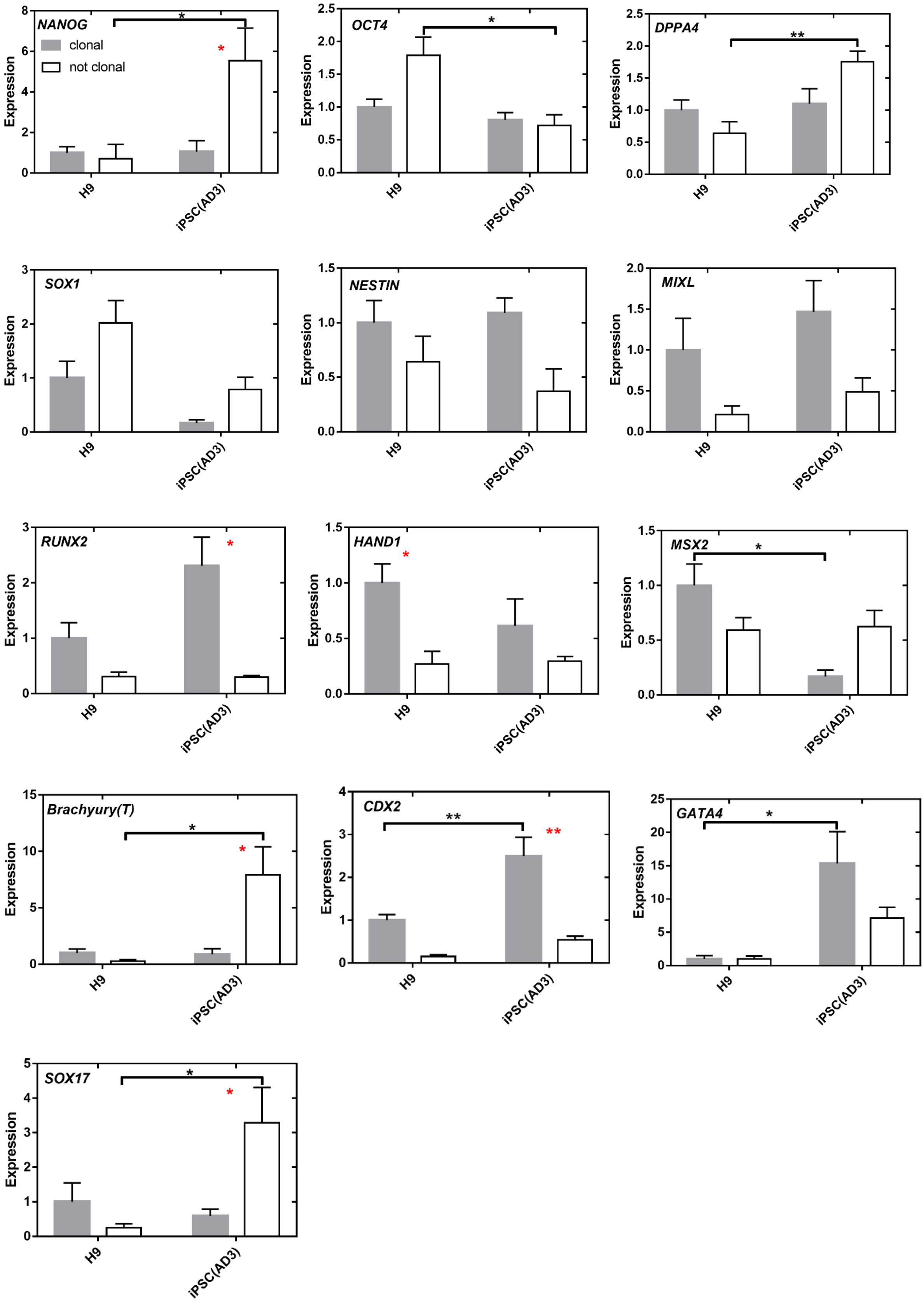

Figure S5. Expression of 13 genes (pluripotency marker genes: *NANOG, OCT4, DPPA4;* ectoderm marker genes: *SOX1, NESTIN*; mesoderm/mesendoderm marker genes: *RUNX2*, *MIXL1, MSX2, HAND1, BRACHYURY(T);* endoderm marker genes: *CDX2, GATA4, SOX17*) in differentiating embryonic bodies derived from the hESC line H9 and hiPSC line AD3, measured in a clonal (gray) and nonclonal (white) colony of each line. Data are presented as mean ± SEM. *, $P < 0,05$; **, $P < 0,01$. Red asterisks point out the comparison of gene expression between clonal and nonclonal colonies, black asterisks point out the comparison between two different cell lines.

Text S1. Classification with manual cross-validation

Classification models in the main text were trained and validated using the Wolfram Mathematica function *Classify* with the default ‘ValidationSet’ option. In this form, the function performs the training on a data with automatic cross-validation, in which validation sets are automatically sampled from the data during the training process, so the user does not need to manually split the data into training and validation datasets. As a control, we performed computational experiments on classification on the cellular data with the manual cross-validation. First, we randomly selected 10% of the cellular data as the test dataset. Second, we randomly split the rest of the data into a training dataset (80% of the split data) and validation dataset (20%), where the validation dataset was aimed to evaluate the model performance during training. The proportion of cells from each cell line in the data remained the same across all data subsets considered. We repeated the last procedure 50 times, so at the output we had one test dataset and 50 pairs of training and validation datasets. We used each pair of these training and validation datasets to retrain the classification models using either four morphological parameters (‘full model’) or the two best parameters (Perimeter and Shape factor; ‘minimal model’) as predictors, by running (50 times) the following command in Mathematica:

Classify[*training_set*, Method → “NeuralNetwork”, ValidationSet → *validation_set*, PerformanceGoal → "Quality"],

where *training_set* is one of the 50 training datasets, *validation_set* is the corresponding validation set used to validate the model performance during the training, and all other options were the same as in the computational experiments used to train the classification models reported in the main text. After obtaining 50 classification models resulted from the multiple running of this command, we calculated the classification accuracy of all models on their training and validation datasets and on the test dataset (Figure S6).

Figure S6 shows that the values of classification accuracy calculated on all data subsets are, on average, very close to each other and very close to the accuracy values reported in the text. Accuracy on the validation sets was 69±2% for the full model as compared to 69±3% reported in the main text, and 67±2% for the minimal model compared to 68±3% reported in the main text. A statistically significant ($P < 0.05$) difference between accuracy on different datasets was recorded only for the training datasets vs. the validation and test datasets for the minimal model (Figure S6), but the absolute values of the average accuracy on these datasets differed from each other by only one percent, which allows us to consider the slight overtraining observed for the minimal model as inessential. Therefore, we conclude that the model training procedure with the default options for validation that was used in the main text produced reliable results.

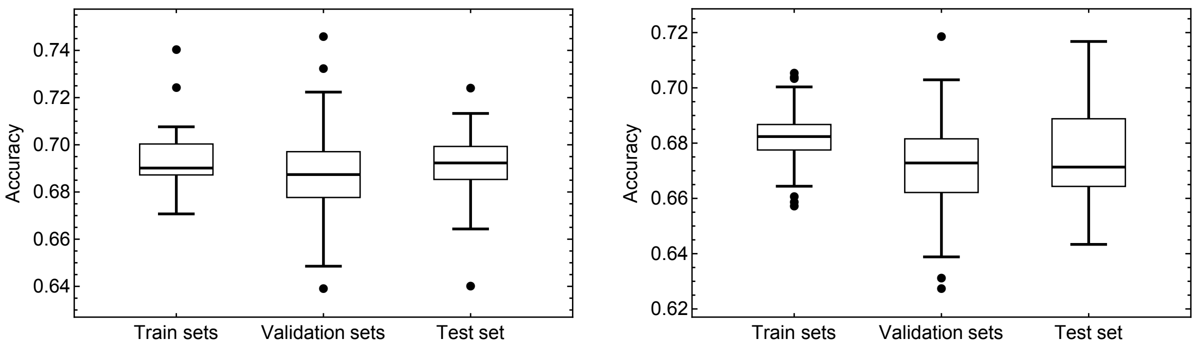

Figure S6. Classification accuracy values of the full (left panel) and minimal (right panel) models from the computational experiments with the manual cross-validation, obtained on the train, validation, and test datasets. The average accuracy ± SD for the full model: 69±1% (training datasets), 69±2% (validation datasets), and 69±1% (test dataset). The average accuracy ± SD for the minimal model: 68±1% (training datasets), 67±2% (validation datasets), and 68±2% (test dataset). Mann–Whitney test for the difference between accuracy on different datasets: train vs. validation, $P = 0.11$ (full model) and $P = 0.002$ (minimal model); train vs. test, $P = 0.99$ (full model) and $P = 0.02$ (minimal model); validation vs. test, $P = 0.17$ (full model) and $P = 0.48$ (minimal model).

Table S1. List of qRT-PCR primers.

	Gene	Forward Primer	Reverse Primer
Pluripotency marker genes	DNMT3B	GGA GAC TCA TTG GAG GAC CA	ACG ACG CAC CTT CGA CTT AT
	SALL4	AGC ACA TCA ACT CGG AGG AG	CCT GGG TGG TTC ACT GGA G
	IGF1R	AGG GGT TTG TGA TCC ACG AC	TGC CCT TGA AGA TGG TGC AT
	CD9	AAG TTA GCC CTC ACC ATG CC	CTG AGA GTC GAA TCG GAG CC
	DPPA4	TGC CCT AAG AAA AAG GCA GA	CTC AGC TTC AAT TGT TGG CA
	OCT4	GAG AAC CGA GTG AGA GGC AAC C	CAT AGT CGC TGC TTG ATC GCT TG
	NANOG	AAT ACC TCA GCC TCC AGC AGA TG	TGC GTC ACA CCA TTG CTA TTC TTC
	REX1	TGT CCT CAG GCT GGG TAG TC	TGA TTT TCT GCC GTA TGC AA
	SOX2	TTG TTC GAT CCC AAC TTT CC	ACA TGG ATT CTC GGC AGA CT
	KLF4	TTA CCA AGA GCT CAT GCC ACC	GCG AAT TTC CAT CCA CAG CC
Ectoderm marker genes	SOX1	GGA ATG GGA GGA CAG GAT TT	ACT TTT ATT TCT CGG CCC GT
	NESTIN	GAGAGGGAGGACAAAGTCCC	CCA CTT CCT CAG ACT GCT CC
Mesoderm/ mesendoderm marker genes	RUNX2	CCG CCT CAG TGA TTT AGG GC	GGG TCT GTA ATC TGA CTC TGT CC
	MIXL	GAGACTTGGCACGCCTGT	GGT ACC CCG ACA TCC ACT T
	HAND1	ACC AGC TAC ATC GCC TAC CTG ATG	TCC CTA TTA ACG CCG CTC CAT
	MSX2	TGG ATG CAG GAA CCC GG	AGG GCT CAT ATG TCT TGG CG
	T	CAG TGG CAG TCT CAG GTT AAG AAG GA	CGC TAC TGC AGG TGT GAG CAA
Endoderm marker genes	CDX2	CTC GGC AGC CAA GTG AAA AC	CTC CTT TGC TCT GCG GTT CT
	SOX17	CGC ACG GAA TTT GAA CAG TA	GGA TCA GGG ACC TGT CAC AC
	GATA4	TCC AAA CCA GAA AAC GGA AG	AAG GCT CTC ACT GCC TGA AG
Housekeeping genes	RN18S	GAA ACT GCG AAT GGC TCA TTA A	GAA TTA CCA CAG TTA TCC AAG TAG GA
	RPLI3A	CCT GGA GGA GAA GAG GAA AGA GA	TTG AGG ACC TCT GTG TAT TTG TCA A
	GAPDH	TGC ACC ACC AAC TGC TTA GC	GGC ATG GAC TGT GGT CAT GAG
